# Deep-fried oil consumption in rats impairs glycerolipid metabolism, gut histology and microbiota structure

**DOI:** 10.1186/s12944-016-0252-1

**Published:** 2016-04-28

**Authors:** Zhongkai Zhou, Yuyang Wang, Yumei Jiang, Yongjia Diao, Padraig Strappe, Paul Prenzler, Jamie Ayton, Chris Blanchard

**Affiliations:** Key Laboratory of Food Nutrition and Safety, Ministry of Education, Tianjin University of Science and Technology, Tianjin, 300457 China; ARC Industrial Transformation Training Centre for Functional Grains, Charles Sturt University, Wagga Wagga, NSW 2650 Australia; NSW Department of Primary Industries, Agriculture Institute, Wagga Wagga, NSW 2650 Australia; School of Food Engineering and Biotechnology, Tianjin University of Science and Technology, Tianjin, 300457 China

**Keywords:** Deep-frying oil, Microbiota, Histology, Metabolism, Kegg pathway

## Abstract

**Background:**

Deep frying in oil is a popular cooking method around the world. However, the safety of deep-fried edible oil, which is ingested with fried food, is a concern, because the oil is exposed continuously to be re-used at a high temperature, leading to a number of well-known chemical reactions. Thus, this study investigates the changes in energy metabolism, colon histology and gut microbiota in rats following deep-fried oil consumption and explores the mechanisms involved in above alterations.

**Methods:**

Deep-fried oil was prepared following a published method. Adult male Wistar rats were randomly divided into three groups (n = 8/group). Group 1: basal diet without extra oil consumption (control group); Group 2: basal diet supplemented with non-heated canola oil (NEO group); Group 3: basal diet supplemented with deep-fried canola oil (DFEO group). One point five milliliters (1.5 mL) of non-heated or heated oil were fed by oral gavage using a feeding needle once daily for 6 consecutive weeks. Effect of DFEO on rats body weight, KEGG pathway regarding lipids metabolism, gut histology and gut microbiota were analyzed using techniques of RNA sequencing, HiSeq Illumina sequencing platform, etc.

**Results:**

Among the three groups, DFEO diet resulted in a lowest rat body weight. Metabolic pathway analysis showed 13 significantly enriched KEGG pathways in Control versus NEO group, and the majority of these were linked to carbohydrate, lipid and amino acid metabolisms. Comparison of NEO group versus DFEO group, highlighted significantly enriched functional pathways were mainly associated with chronic diseases. Among them, only one metabolism pathway (i.e. glycerolipid metabolism pathway) was found to be significantly enriched, indicating that inhibition of this metabolism pathway (glycerolipid metabolism) may be a response to the reduction in energy metabolism in the rats of DFEO group. Related gene analysis indicated that the down-regulation of *Lpin1* seems to be highly associated with the inhibition of glycerolipid metabolism pathway. Histological analysis of gastrointestinal tract demonstrated several changes induced by DFEO on intestinal mucosa with associated destruction of endocrine tissue and the evidence of inflammation. Microbiota data showed that rats in DFEO group had the lowest proportion of *Prevotella* and the highest proportion of *Bacteroides* among the three groups. In particular, rats in DFEO group were characterized with higher presence of *Allobaculum* (*Firmicutes*), but not in control and NEO groups.

**Conclusion:**

This study investigated the negative effect of DFEO on health, in which DFEO could impair glycerolipid metabolism, destroy gut histological structure and unbalance microbiota profile. More importantly, this is the first attempt to reveal the mechanism involved in these changes, which may provide the guideline for designing health diet.

**Electronic supplementary material:**

The online version of this article (doi:10.1186/s12944-016-0252-1) contains supplementary material, which is available to authorized users.

## Background

Microbes present in the gastrointestinal tract have a tremendous effect on host physiology, including protection from pathogenic microorganisms [1], obtaining nutrients and energy and fermentation of non-digestible carbohydrates [2]. The structure of this microbial community contributes to the host in terms of modulating the immune system, metabolism and combating infection [3–5]. The composition and activities of intestinal bacteria may change in response to a number of environmental or lifestyle variables, one of the most important being diet [6]. In fact, it has been reported that dietary alterations are responsible for 57 % of the gut microbiota’s entire variation, whereas genetic background may only contribute to 12 % [7]. Furthermore, the disruption of the normal host microbial community can contribute to the development of chronic diseases such as obesity, diabetes and irritable bowel disease [8]. Diet is a major determining factor of the gut microbiota [3, 9, 10]. For example, previous study found that the ratio of *Bacteroidetes* to *Firmicutes* increased in the gut of people consuming either fat or carbohydrate restricted diets, and a higher ratio was associated with greater weight loss [11].

Deep frying in oil is one of the most popular cooking methods worldwide and provides an appealing flavor, color and texture to the foods. However, foods fried between 150 and 180 °C have been shown to absorb up to 8 to 25 % of the oil [12], and this is dependent on food types, oil properties and cooking methods, indicating that frying oil is becoming a major dietary component in the food formula. Thus, the safety of deep-fried edible oil (DFEO) is a concern because it is ingested with fried food. The continuous cooking at high temperature (160 ~ 190 °C) in the presence of air and moisture leads to a number of chemical reactions (e.g. oxidation, hydrolysis and polymerization, etc.) [13, 14]. These reactions alter the composition of the oil with the production of various types of oxidative products [15], such as oxidized fatty acids, polar compounds and polymeric products [16–18]. Previous studies confirmed that the consumption of deep oil fried foods contributes to chronic diseases such as obesity, atherosclerosis, hypertension and diabetes [19–21]. Furthermore, the intake of over-cooked edible oil could elevate blood pressure and impair vasorelaxiation in rats which maybe attribute to endothelial dysfunction [20], and the studies have also described the potential links between deep-fried oil and the increased risk of prostate cancer [22]. Previous work focused mainly on physical and chemical changes of the oils occurred during deep-frying process. To date there has not been sufficient studies to directly or indirectly investigate the ingestion of deep-fried edible oil (DFEO) on the changes in gut microflora. Therefore, the purpose of this study was to evaluate potential toxicity of DFEO on gastrointestinal environment through the regulation of gut microbiota structure. Moreover, the effect of DFEO consumption on energy metabolism, in particular lipid metabolism, was also investigated. To the best of our knowledge, this is the first attempt to reveal the potential links between energy metabolism and gut microbiota community structure.

## Results

### Effect of consumption of different oil types on body weight of rats

**Fig. 1:**
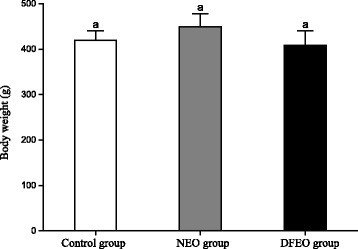
Effect of different types oil consumption on animal body weight. Control: basal diet without extra oil consumption; NEO: basal diet plus non-heated canola oil; DFEO: basal diet plus deep-fried canola oil

Of the three experimental groups, it was expected to see that rats in NEO group had the highest body weight (Fig. 1) due to the extra oil consumption in the diet, followed by the rats in control group. In contrast, the feeding of DFEO resulted in rats having the lowest body weight among the three groups although the difference was not significant.

### Pathway significant enrichment analysis

KEGG pathway mapping was implemented based on KEGG ontology (KO) terms. KEGG ontology assignments were used to classify the functional annotations of the identified genes to further understand the biological function (Table 1). A total of 13 significantly enriched KEGG pathways in control versus NEO group was determined, in which the majority of these pathways were linked to the metabolism of carbohydrate, lipids and amino acids based on enriched signaling pathways of “pentose and glucuronate interconversions”, “steroid hormone biosynthesis” and “histidine metabolism”, respectively (control versus NEO group in Table 1). This result may suggest the consumption of non-heated oil accelerated energy metabolism, which led to an increase in rat body weight (Table 1). Meanwhile, there were 47 significantly enriched functional pathways related to the gene regulation induced by different treatments in NEO versus DFEO group, which are mainly associated with infections and cancers, such as microRNA regulation and transcriptional misregulation in cancer. More importantly, there was only one significantly enriched metabolism pathway, i.e. glycerolipid metabolism, detected in NEO versus DFEO group (Table 1). To the best of our knowledge, this is the first report to clarify the key pathway involved in the effect of DFEO consumption on lipids metabolism.

**Table 1:** Significantly enriched functional pathways of gene regulation induced by different treatments between groups

Pathway name	Fold enrichment	*P* value	FDR	Pathway id
NC versus FO				
ABC transporters	7.271852	0.002065	0.215471	rno02010
Histidine metabolism	10.1964	0.002952	0.215471	rno00340
Circadian rhythm	8.685824	0.004692	0.228324	rno04710
Steroid hormone biosynthesis	5.211494	0.006869	0.236681	rno00140
p53 signaling pathway	4.963328	0.008141	0.236681	rno04115
Alanine, aspartate and glutamate metabolism	6.700493	0.009727	0.236681	rno00250
Prolactin signaling pathway	4.28342	0.013457	0.280675	rno04917
Cell cycle	3.31239	0.016186	0.2954	rno04110
Arginine and proline metabolism	4.187808	0.033798	0.493447	rno00330
Non-small cell lung cancer	4.187808	0.033798	0.493447	rno05223
Retinol metabolism	3.844545	0.041883	0.515193	rno00830
Transcriptional misregulation in cancer	2.521691	0.044352	0.515193	rno05202
Pentose and glucuronate interconversions	5.790549	0.045873	0.515193	rno00040
FO versus DO				
Glycerolipid metabolism	4.186519	0.0146	0.090976	rno00561

Analysis of the genes related to KEGG on glucose and lipid metabolisms indicated that there were 5 genes (*Agpat9*, *Lpin1*, *Akr1b1*, *Tkfc*) involved in the regulation of glycerolipid metabolism in NEO versus DFEO group (Table 2). Among them, the down-regulation of gene of *Lpin1*, with the lowest *P* vale of 5.00 × 10^−5^, seems to play the most key role for the inhibition of lipids metabolism.

**Table 2:** Genes related to KEGG on glucose and lipid metabolism

Group	KEGG pathway	Gene id	Gene	log_2_ (fold change)	*P* value	q value
Control vs NEO	Histidine metabolism (rno00340)	ENSRNOG00000011497	*Aldh1b1*	−2.37141	0.00095	0.154268
		ENSRNOG00000010262	*Hdc*	−1.81999	0.00035	0.07551
		ENSRNOG00000019659	*Aspa*	−1.11487	0.03735	0.998315
	Steroid hormone biosynthesis (rno00140)	ENSRNOG00000013982	*Hsd17b2*	1.09138	0.0006	0.114699
		ENSRNOG00000020035	*Cyp17a1*	−1.40388	5.00E-05	0.015102
		ENSRNOG00000021405	*LOC100361547*	1.779	5.00E-05	0.015102
		ENSRNOG00000024016	*Cyp2c6v1*	1.6773	0.02205	0.998315
	Alanine, aspartate and glutamate metabolism (rno00250)	ENSRNOG00000016356	*Got1*	−1.61798	5.00E-05	0.015102
		ENSRNOG00000017821	*Agxt2*	1.24264	0.0001	0.028494
		ENSRNOG00000019659	*Aspa*	−1.11487	0.03735	0.998315
	Arginine and proline metabolism (rno00330)	ENSRNOG00000011497	*Aldh1b1*	−2.37141	0.00095	0.154268
		ENSRNOG00000016356	*Got1*	−1.61798	5.00E-05	0.015102
		ENSRNOG00000016807	*Oat*	−1.11901	0.0008	0.140484
	Pentose and glucuronate interconversions (rno00040)	ENSRNOG00000009875	*Akr1b7*	−2.04813	0.02795	0.998315
		ENSRNOG00000011497	*Aldh1b1*	−2.37141	0.00095	0.154268
NEO vs DFEO	Glycerolipid metabolism (rno00561)	ENSRNOG00000002159	*Agpat9*	−1.18199	0.01145	0.692947
		ENSRNOG00000004377	*Lpin1*	−1.33227	5.00E-05	0.017883
		ENSRNOG00000009513	*Akr1b1*	−1.00862	0.0311	0.997737
		ENSRNOG00000020704	*Tkfc*	1.02365	0.00135	0.247604

### Histological study

**Fig. 2:**
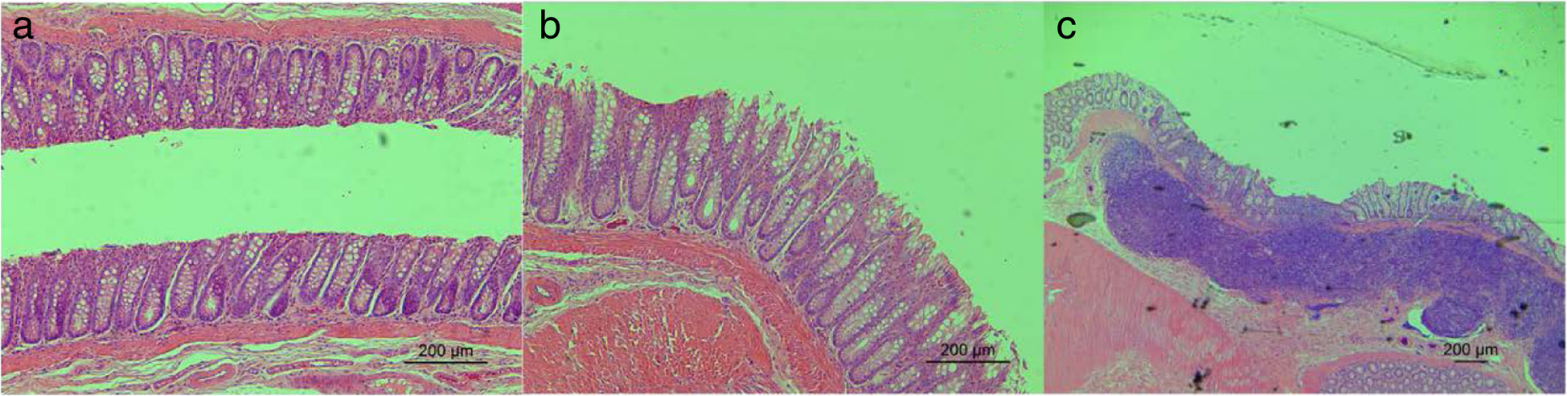
Histological analysis of colon tissue in rats fed the normal diet (control, 2**a**, HE, ×100), basal diet plus non-heated canola oil (NEO, 2**b**, HE, ×100), and basal diet plus deep-fried oil (DFEO, 2**c**, HE, ×40)

Rats in the control group displayed a normal colon histological structure (Fig. 2a), and rats in the NEO group did not show any significant changes in the colon histological structure (Fig. 2b) compared to the control group. However, rats in DFEO group showed characteristics of colon walls being diffuse and infiltrated with lymphocytes (inflammation), which was observed in 2/8 cases in DFEO group (Fig. 2c), and in one of them, the normal glandular structure disappeared, replaced by connective tissue.

### Effects of different oil consumptions on bacterial community structure

A total of 1,034,683 effective tags and 19,418 OTUs were obtained through Illumina HiSeq analysis. Rationality of sequencing data was evaluated by rarefaction curve (Additional file 1: Figure S1). It was observed that the rarefaction curve tended to be flat when the sequence number increased to 20,000 (Additional file 1: Figure S1), indicating that the amount of sequencing data was reasonable. Moreover, all samples in this study had at least 30,000 sequences.

**Fig. 3:**
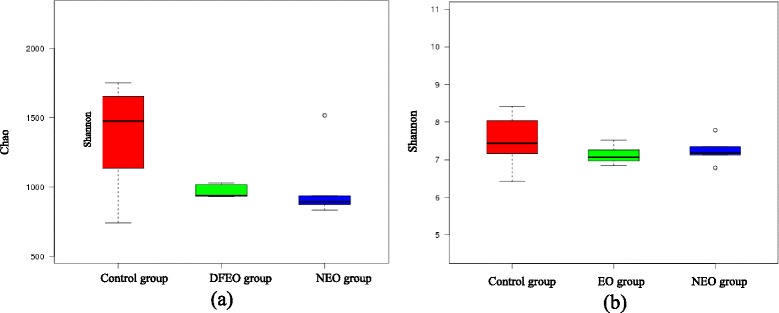
Chao **a** and Shannon **b** indices showing bacterial community richness and diversity in each group. Control: basal diet without extra oil consumption; NEO: basal diet plus unheated canola oil; DFEO: basal diet plus deep-fried canola oil

Among the three groups, samples from control group had the most tags and OTUs, and the OTUs of the other two groups significantly declined in comparison with the control group. From high to low, it was control, DFEO, and NEO group (Additional file 1: Figure S2). This result was verified by Chao index which is used to evaluate intestinal community richness. A larger Chao index is associated with a higher community richness. From high to low, it was control, DFEO and NEO (Fig. 3a). Meanwhile, the intestinal bacterial community diversity was also evaluated using the Shannon index (Fig. 3b). The greater the Shannon index, the higher the community diversity. Thus, the current data showed that samples from control group had the highest bacterial community diversity, followed by NEO group, while the lowest bacterial community diversity was seen in the rats of DFEO group in this study. This result further suggested that diet was a major determinant of microbial richness and the extra oil consumption in the diet obviously reduced gut bacterial richness and diversity.

**Fig. 4:**
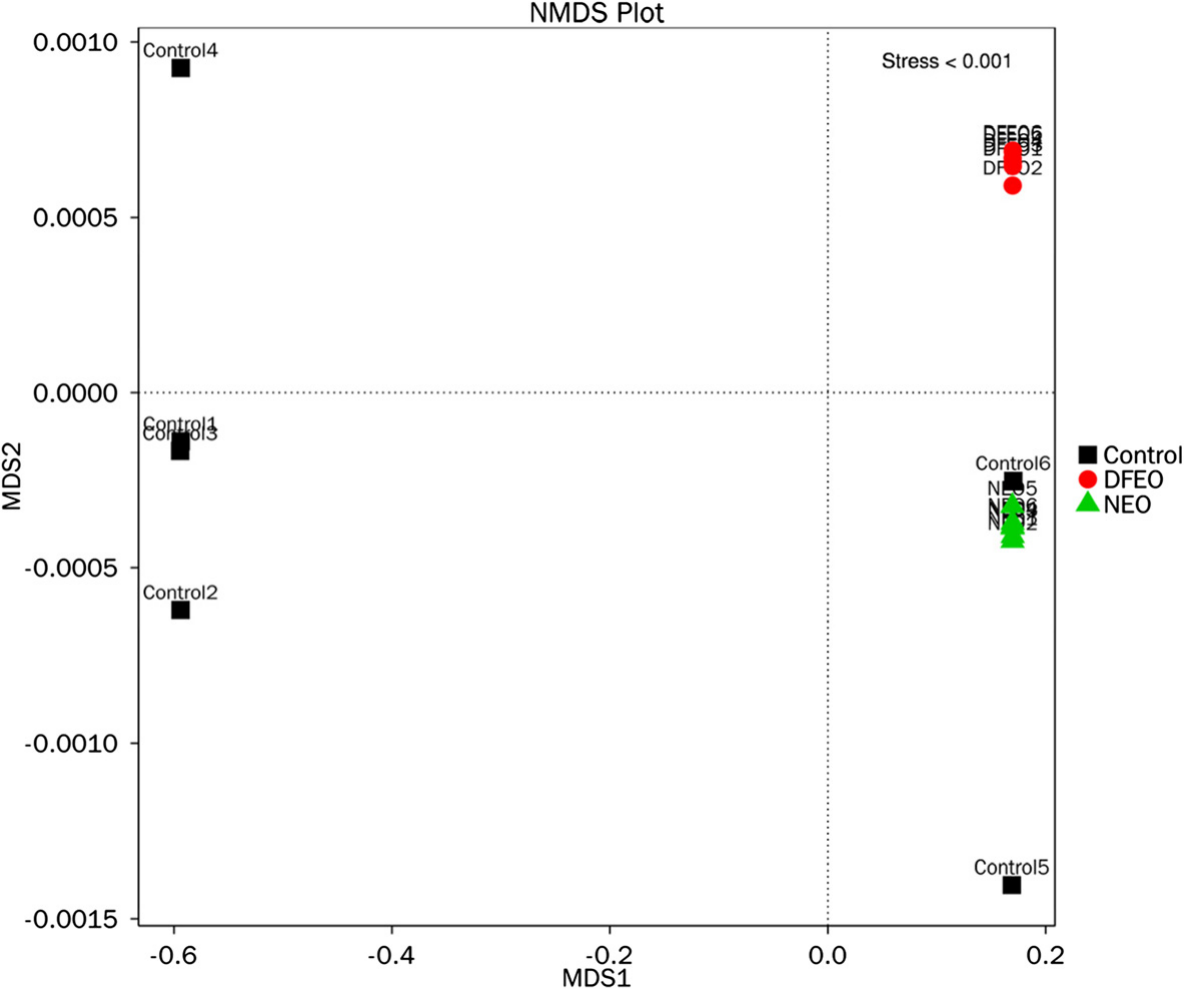
NMDS analysis showing difference in terms of species in fecal samples. Beta diversity was analyzed on unweighted UniFrac. Control: basal diet without extra oil consumption; NEO: basal diet plus unheated canola oil; DFEO: basal diet plus deep-fried canola oil

**Fig. 5:**
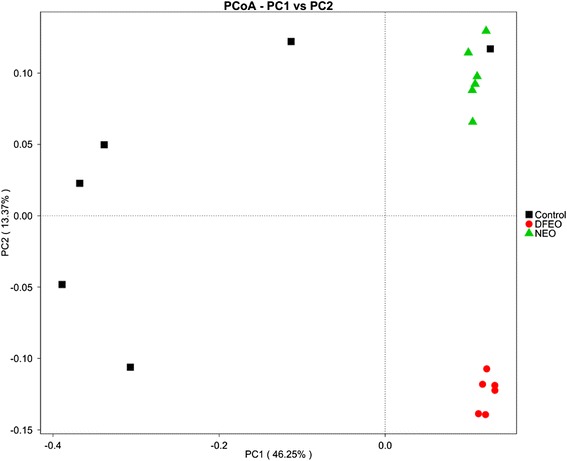
PCoA analysis showing difference in terms of species in fecal samples. Beta diversity was on weighted UniFrac. Control: basal diet without extra oil consumption; NEO: basal diet plus unheated canola oil; DFEO: basal diet plus deep-fried canola oil

**Fig. 6:**
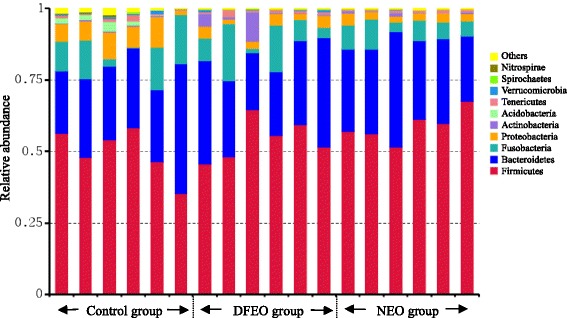
The relative abundance of microbial species at the phylum level in the feces of rats. Control: basal diet without extra oil consumption; NEO: basal diet plus unheated canola oil; DFEO: basal diet plus deep-fried canola oil

The overall bacterial community composition was compared using weighted (NMDS) and unweighted UniFrac (PCoA) distance matrices (Fig. 4, Fig. 5). Both PCoA and NMDS showed that all the samples in the three groups were trisected. Figure 6 shows that the samples in the control group were separated from the other two groups along PC1, which represented 46.25 % of the total variation. Because the feeding conditions of the three groups were identical, the distinct clustering along PC1 reflects that the administration of oil dramatically influenced the bacterial community composition. The point distance between DFEO and NEO groups was adjacent and separated along PC2, which represented 13.37 % of the total variation. This may suggest that the overall bacterial community composition of these two groups still showed some similarity, which may be related to the dominant roles associated with the major oil components. Furthermore, MRPP analysis was carried out to further compare the differences between the groups and within in each individual group (Additional file 1: Table S2), and it shows that the difference between any two groups was greater than the difference within an individual group. These results verified the PCoA and NMDS analyses, and demonstrated that diet has an important influence on the composition of gut microbiota and the groupings are reasonable for the current study.

At phylum level, the proportion of *Firmicutes* increased in both NEO and DFEO groups comparing with the control group (Fig. 6). Meanwhile, this study also found that the proportion of *Bacteroidetes* increased in the NEO group and decreased in DFEO group compared with the control group although the difference was not significant. This bacterial profiling pattern influenced by diet seems to be consistent with that of animal body weight as revealed in Fig. 1.

**Fig. 7:**
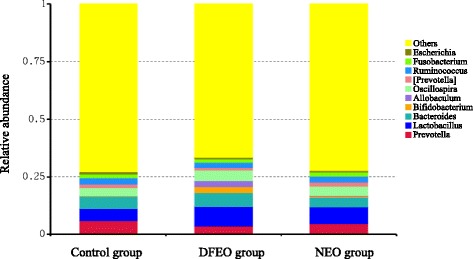
The relative abundance of microbial species at the genus level in the feces of rats. Control: basal diet without extra oil consumption; NEO: basal diet plus unheated canola oil; DFEO: basal diet plus deep-fried canola oil

**Fig. 8:**
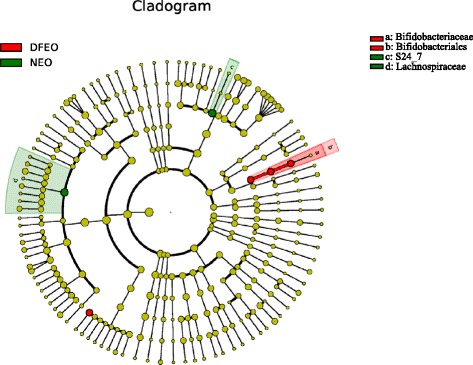
Cladogram representing taxa enriched in fecal samples of rats, detected by the LEfSe tool. NEO: basal diet plus unheated canola oil; DFEO: basal diet plus deep-fried canola oil

At genus level, *Prevotella* was the most prevalent genus in the control group (0.05848), and *Lactobacillus* was the most prevalent genus in the NEO and DFEO groups (0.070665 and 0.0839, respectively) (Fig. 7). Among the top 10 genuses, the relative abundance of *Prevotella* (*Bacteroidetes)* and *Ruminococcus* (*Firmicutes*) was lower both in DFEO and NEO groups compared with the control group. Moreover, this study also found that rats in DFEO group had the lowest proportion of *Prevotella* among the three groups (Fig. 8), and this reduction of *Prevotella* in DFEO group was significant, indicating that DFEO consumption further inhibits the growth of *Prevotella*.

In comparison to the control group, the proportion of some genus increased in NEO and DFEO groups. For example, rats in DFEO group had a highest proportion of *Lactobacillus* (*Firmicutes*), *Bacteroides* (*Bacteroidetes)*, *Oscillospira* (*Firmicutes*), Bifidobacterium (Actinobacteria). In particular, rats in DFEO group were characterized with high presence of *Allobaculum* (*Firmicutes*), but not for control and NEO groups (Fig. 7). Except for DFEO group, *Lactobacillus* was also the most abundant genus in the rats of NEO group.

It has to be mentioned that, in this study, flatulence was observed in the rats of DFEO group, and two rats died caused by the DFEO toxicity. This is consistent with the increased number of *Bacteroides* in DFEO group, which can exacerbate abscesses and other infections. However, the mechanism of flatulence needs further study. For example, *Clostridium perfringens* was also found to be a statistically significant species among the groups. In addition, *Bifidobacteriales. S24_7* and *Bacteroidiales* were associated with DFEO, and *Veillonellaceae* and *Clostridia* significantly increased in the NEO group. Furthermore, Cladogram (Fig. 8) also showed that the proportions of *Bifidobacteriaceae* and *Bifidobacteriales* were highly associated with DFEO, whereas S24_7 and *Lachnospiraceae* were characterized in the rats of NEO group.

## Discussion

This study found that DFEO consumption led the rats to having the lightest body weight, which may indicate that the toxic components from DFEO have a negative impact on the metabolism and thus decrease rat weight gain. The growth response of rats fed with DFEO in this study is consistent with the findings of López et al. who reported that diet containing deep-fried oil led to animals having less weight gain [23]. Considering that the significantly enriched KEGG pathways in control versus NEO groups are mainly associated with metabolism disorder, this study further provided the evidence that the repeated consumption of over-cooked edible oil may increase the risk of chronic diseases, which is consistent with other studies [22, 24]. In particular, only one significantly enriched metabolism KEGG pathway, glycerolipid metabolism, was detected in NEO versus DFEO group (Table 1), which may provide a better understanding of the mechanism involved in DFEO influence on lipids metabolism, because glycerolipid (GL)/free fatty acid (FFA) cycle is referred to as a “futile” cycle, in which it involves continuous formation and hydrolysis of GL with the release of energy at the expense of ATP [25]. Thus, this study may indicate that the DFEO toxicity impairs energy metabolism, in particular through regulating the pathway of glycerolipid metabolism. The inhibition of the pathway regarding glycerolipid metabolism led to the reduction in energy absorption in rats of DFEO group, which subsequently influence rats growing performance. Four genes expressions related to this pathway were also investigated, and the down-regulation of these genes, in particular, one of them, Lpin1, may play the key roles for regulating the energy metabolism, because gene *Lpin1* encodes a magnesium-ion-dependent phosphatidic acid phosphohydrolase enzyme that catalyzes the penultimate step in triglyceride synthesis including the dephosphorylation of phosphatidic acid to yield diacylglycerol [26]. The finding in the lipids metabolism in this study is consistent with the decreased body weight of the rats in DFEO group compared to the rats in other two groups.

The influence of DFEO consumption on rats growing performance was further investigated from the damage induced by DFEO on intestinal mucosa, including directly destroy of gland tissue structure. These histological structural changes may be associated with direct damage induced by the toxic compounds in DFEO which may indirectly influence changes in the gut microbial profile. The bacterial profile study showed that more oil consumption in the diet led to a reduction of both Chao and Shannon indices, indicating the lower bacterial community richness and diversity in rats of NEO and DFEO groups. This was consistent with a previous study that ingestion of a high-fat diet in rats alters the gut microbiota, leading to a decrease in total bacterial density and diversity [27, 28], suggesting that diet is one of the most important factors for influencing the composition of gut microbiota. Furthermore, the lowest bacterial community diversity was found in the rats of DFEO group in this study, which further suggests that the chemical structure in DFEO may also significantly influence microbial structure. In general, the higher the microbial diversity, the more stable the ecosystem. Thus, from this study it might indicate that the consumption of DFEO reduced the intestinal microbial diversity, which may contribute to its potential negative effect on gut.

At phylum level, *Firmicutes* and *Bacteroidetes* were most dominant in all three groups, which were in agreement with previous studies [11, 29], and the oil consumption achieved a higher proportion of *Firmicutes*, which agrees with the previous reports that *Firmicutes* increased in fecal samples in mice following the feed of high fat diets [6, 11]. However, the decreased proportion of *Bacteroidetes* in DFEO group and increased in the NEO group compared with the control group may directly influence rats growing performance, and these bacterial patterns were consistent with their corresponding body weights found in this study.

At the genus level, *Prevotella*, this study found the proportion of *Prevotella* was decreased in the rats of NEO and DFEO groups, indicating that oil present in the diet may inhibit the proliferation of *Prevotella*, and induce the proliferation of bacteria which take part in the degradation and utilization of fat [30]. This result may be associated with a function of *Prevotella*, which has been implicated in the degradation and utilization of carbohydrate and simple sugars, and strongly related with long-term diet [31]. However, the lowest proportion of *Prevotella* in DFEO group may imply more complicated mechanism involved in this the interactions between diet and gut bacteria.

More importantly, rats in DFEO group were characterized with higher existence of genus *Allobaculum* (*Firmicutes*), but not found in control and NEO groups (Fig. 8). This difference may be used to characterize the gut microbiota in rats of DFEO group. Meanwhile, previous studies have reported that a high level of dietary fat is accompanied with a significant reduction in the abundance of lactic acid bacteria [32, 33]. However, in this study, the amount of *Lactobacilli* increased both in NEO and DFEO groups, especially in DFEO group. The plausible interpretation of these results may be that: the long-term administration of DFEO induced an immune stress on the intestine, which contributed to a loss of immune homeostasis. There is a direct relationship between the intestinal flora and the immune state of animals [34]. The immune system may have been primed to resist potential chronic stressors (e.g. various types of oxidative products in DFEO) by activating an increase of beneficial bacteria, such as *Lactobacillus* and *Bifidobacterium*. Meanwhile, the extra administration of oil caused the compensatory proliferation of *Lactobacillus*, as *Lactobacillus* can down-regulate lipogenesis and up-regulate lipolysis and fatty acid oxidation [35]. Alternatively, it has been reported that the longer the frying time, a lower pH is achieved, and the activity of H^+^ of DFEO becomes 100 times higher than that of non-heated oil [36]. Therefore, the administration of DFEO may induce a reduction of the gut pH value, in which the low pH environment further promotes the growth of *Lactobacillus* and *Bifidobacterium*. Nevertheless, the long-term effect, especially deep-fried oil needs to be further investigated. Considering the complication of reactions between diet and gut microbiotia, and in particular, there are no related studies describing different influences of non-heated and deep-fried oils on gut microbiota, the indicative action of these microbiota needs to be further verified.

## Conclusions

Consumption of oil following deep-frying process had a different impact on body weight, lipid metabolism, gut microbiota and histological properties. This study found that the rats with feeding of DFEO had the lowest body weight among the three groups. More importantly, this is the first report to show that there was only one significantly enriched KEGG pathway, glycerolipid metabolism, detected in NEO versus DFEO group, indicating that the DFEO toxicity impairs energy metabolism, in particular through the regulation of the pathway of glycerolipid metabolism. The inhibition of the pathway regarding glycerolipid metabolism was highly down-regulated by gene of *Lpin1*, and this change may be associated with the decreased body weight of the rats in DFEO group. Compared with non-heated oil, the consumption of deep-fried oil has potential harmful effects on gut histological structure. As a pro-inflammatory potential, the consumption of DFEO was found to be associated with a decrease in *Prevotella* and an increase in *Bacteroides*. In particular, rats in DFEO group were also characterized with higher presence of *Allobaculum* (*Firmicutes*). Furthermore, the increased proportion of *Lactobacillus* and *Bifidobacterium* in the rats of DFEO group may be related with an immune stress induced by DFEO-containing diet.

## Methods

### Materials

Non-heat edible oil (fresh canola oil, NEO) was purchased from a local supermarket. Deep-fried edible oil (canola oil following deep-frying process, DFEO) was prepared as described previously [37]. In Brief, fresh canola oil was heated at 190 ± 5 °C for 4 intermittent days (8 h each day) for a total of 32 h. Fresh canola oil (7 L) was poured into an iron saucepan with a bore of 45 cm and a depth of 20 cm, and 100 g of chicken nuggets, potato chips, bread pieces, or fish were fried for 4 or 2 min, respectively, in succession for a total of 30 min without replenish. Other chemicals were of reagent grade and used as received.

### Animals and diets

Male, 6 weeks old Wistar rats of 295 ± 10 g weight were purchased from the animal house, Chinese Military Medical Science Academy. After 1 week’s adaptive feeding with the basic diet, the rats were randomly divided into three groups. Group 1: basal diet without extra oil consumption (control group); Group 2: basal diet supplemented with non-heated canola oil (NEO group); Group 3: basal diet supplemented with deep-fried canola oil (DFEO group). One point five milliliters (1.5 mL) of either non-heated or heated oil was fed by oral gavage using a feeding needle once daily for 6 consecutive weeks before animals were sacrificed for analysis. Each group had eight animals housed in plastic cages (4 rats/cage) with free access to water and food. The conditions of humidity (55 ± 5 %), light (12/12 h light/dark cycle) and temperature (at 23 °C) were controlled throughout the entire experimental period. The main ingredients of the basal diet (standard rodent chow) are shown in Additional file 1: Table S1.

### Histological study

After dissection of animals, intestinal tissues were removed and fixed in 10 % neutral formalin for 48 h, washed in running tap water for 24 h. The tissues were then dehydrated using 30, 50, 70, 80, 90, 95 and 100 % ethanol, cleared in two changes of xylene, embedded in paraffin (BMJ-III embedding machine, Changzhou Electronic Instrument Factory, Jiangsu, China), and then cut into 5-μm thick sections using a microtome (Leica RM2235; Leica, Heidelberg, Germany). Slides were stained with haematoxylin and eosin (H&E) for histological examination.

### Total RNA extraction and quantitative RT-PCR analysis

After the 6-week experimental trial, rats were dissected immediately with sterile scissors. The liver was removed, weighed, cut into 0.5-cm^3^ pieces, immediately frozen in liquid nitrogen, and then stored at−80 °C before homogenizing for total RNA extraction.

Total RNA was extracted from each liver sample using Trizol Reagent (Invitrogen, Life Technologies, Carlsbad, CA, USA) following the manufacturer’s protocol. Purified poly (A) + mRNA was extracted from the total RNA sample using Oligo (dT) magnetic beads. Total RNA and cDNA syntheses were performed as described below and the resultant cDNA was stored at−20 °C until qRT-PCR analysis, which was carried out in a 20 μL volume containing 2 μM of each primer, 40 ng of cDNA, and 10 μL of SYBR Primix ExTag. Thermal cycling conditions included an initial denaturation step at 95 °C for 5 min, and then 40 cycles of 95 °C for 30 s, 58–60 °C for 30 s and 72 °C for 30 s. Fluorescence was measured at the end of each cycle. The 18S rRNA gene was used as an internal control to normalize target gene expression. Three replicates of each reaction were carried out, and the relative transcript quantity was calculated according to the method of 2-ΔΔCT [38].

### Digital gene expression tag profiling

The mRNA was sheared into short fragments by adding a fragmentation buffer. First-strand cDNA was synthesized from these short poly (A) + mRNA fragments by adding random primers and Superscript II. Buffer, dNTPs, DNA polymerase I, and RNaseH were then added to generate second-strand cDNA. The double-stranded cDNA was end-repaired by adding T4 DNA polymerase, Klenow Enzyme and T4 polynucleotide kinase. This was followed by a single ‘A’ base addition using Klenow 3–5’ exo-polymerase, and then sequencing adapters were ligated to the fragments using DNA ligase. For high-throughput sequencing, the cDNA fragments (PE200) were separated by agarose gel electrophoresis and then sequenced on the Illumina Hiseq™ 2000 platform.

Transcript abundance and differential gene expression were calculated with the program Cufflinks [39]. The *P* value threshold was determined by the false discovery rate (FDR) to account for multiple tests of significance. In this study, a FDR threshold ≤ 0.01 and a Fold change ≥ 2 were considered as significant differences in gene expression.

### Gut microbiota analysis

#### Fecal sample collection, DNA extraction and purification

After 6 weeks feeding, all rats were transferred to fresh sterilized cages and the fresh feces were collected, immediately frozen in liquid nitrogen, and then stored at−80 °C until DNA extraction. Six fecal samples of each group were taken.

Microbial DNA was extracted from 200 mg samples using the E.Z.N.A. DNA Stool Mini Kit (Omega Biotek, Germany) according to the manufacturer’s protocols. For each sample, DNA was extracted in duplicate to avoid bias, and the extracts from the same sample were pooled. DNA purity and concentration were analyzed spectrophotometrically using the e-Spect ES-2 (Malcom, Japan). The extracted DNA was stored at−20 °C until use.

#### PCR amplification of 16S rDNA V4 hypervariable regions

Sequences encompassing V4 16S rDNA hypervariable regions were PCR amplified from DNA samples using fusion primers (515 F and 806R). All PCR reactions were carried out with Phusion® High-Fidelity PCR Master Mix (New England Biolabs). PCR products were analyzed by mixing the same volume of 1X loading buffer (contained SYB green) with PCR product and separated by electrophoresis on 2 % agarose gel. Samples with a bright main band between 400 and 450 bp were chosen for further experiments. PCR products were then purified with a Qiagen Gel Extraction Kit (Qiagen, Germany). Sequencing libraries were generated using TruSeq® DNA PCR-Free Sample Preparation Kit (Illumina, USA) following manufacturer's recommendations and index codes were added. The library quality was assessed on a Qubit@ 2.0 Fluorometer (Thermo Scientific) and Agilent Bioanalyzer 2100 system. Finally, the library was sequenced using an Illumina HiSeq2500 platform and 250 bp paired-end reads were generated.

#### Sequence analysis

All of the raw reads were treated according to the standard protocols and effective Tags were acquired [40–42]. Based on the HiSeq Illumina sequencing platform, the double end sequencing (Paired-End) method was used. Terminal sequencing was constructed by a small fragment library. Sequences analysis was performed by Uparse software (Uparse v7.0.1001). Sequence data were processed by read trimming and identification of V4 sequences, followed by filtering and assigning the operational taxonomic units (OTUs). Sequences with ≥97 % similarity were assigned to the same OTUs. Sample species composition was revealed by OTUs cluster, species annotation and abundance analysis.

The species richness and diversity of microbial communities in different samples were analyzed by Chao and Shannon indices. Sample index was calculated with QIIME (Version 1.7.0) and displayed with R software (Version 2.15.3). Beta diversity analysis was used to comparatively analyze the microbial community diversity in different samples. Beta diversity on both weighted and unweighted unifrac was calculated by QIIME software (Version 1.7.0). The difference between samples in terms of species was shown by PCoA (Principal Co-ordinates Analysis, PCoA) and NMDS (Non-Metric Multi-Dimensional Scaling, NMDS). MRPP analysis was used to compare the differences in community structure within and between the groups.

### Statistical analysis

Body mass was expressed as mean ± SD and analyzed using the Fisher test. *T*-test and LDA Effect and Size analysis were used to determine statistically significant differences in biomarkers and in gut microbiota among the different groups. *P* < 0.05 was considered as significant difference.

### Ethics statement

All animal trial procedures were reported and approved by the Ethical Committee for the Experimental Use of Animals in Center for Drug Safety Evaluation, Tianjin University of Science & Technology (approval No:17/055/MIS).

## Additional file

Additional file 1: Figure S1. Rarefaction Curve showing the rationality of bacterial community in different groups. Control: basal diet without extra oil consumption; NEO: basal diet with unheated canola oil; DFEO: basal diet with deep-fried canola oil. Figure S2. Clustering and annotation of the OTUs of samples. The X-axis is different sample names. The first Y-axis is Tags number, and the second Y-axis is OTUs number. Table S1. Components of basal diets. Table S2. MRPP for difference among control, NEO and DFEO groups. (DOC 198 kb)
