# Supplementary material for: Deep-fried oil consumption in rats impairs glycerolipid metabolism, gut histology and microbiota structure
Source: Lipids Health Dis. 2016 Apr 28;15:86. doi: 10.1186/s12944-016-0252-1 (PMC4848804; doi:10.1186/s12944-016-0252-1)
Supplement: Additional file 1: Figure S1. — Rarefaction Curve showing the rationality of bacterial community in different groups. Control: basal diet without extra oil consumption; NEO: basal diet with unheated canola oil; DFEO: basal diet with deep-fried canola oil. Figure S2. Clustering and annotation of the OTUs of samples. The X-axis is different sample names. The first Y-axis is Tags number, and the second Y-axis is OTUs number. Table S1. Components of basal diets. Table S2. MRPP for difference among control, NEO and DFEO groups. (DOC 198 kb) [file 12944_2016_252_MOESM1_ESM.doc]

**Fig. S1.** Rarefaction Curve showing the rationality of bacterial community in different groups. Control: basal diet without extra oil consumption; NEO: basal diet with unheated canola oil; DFEO: basal diet with deep-fried canola oil.


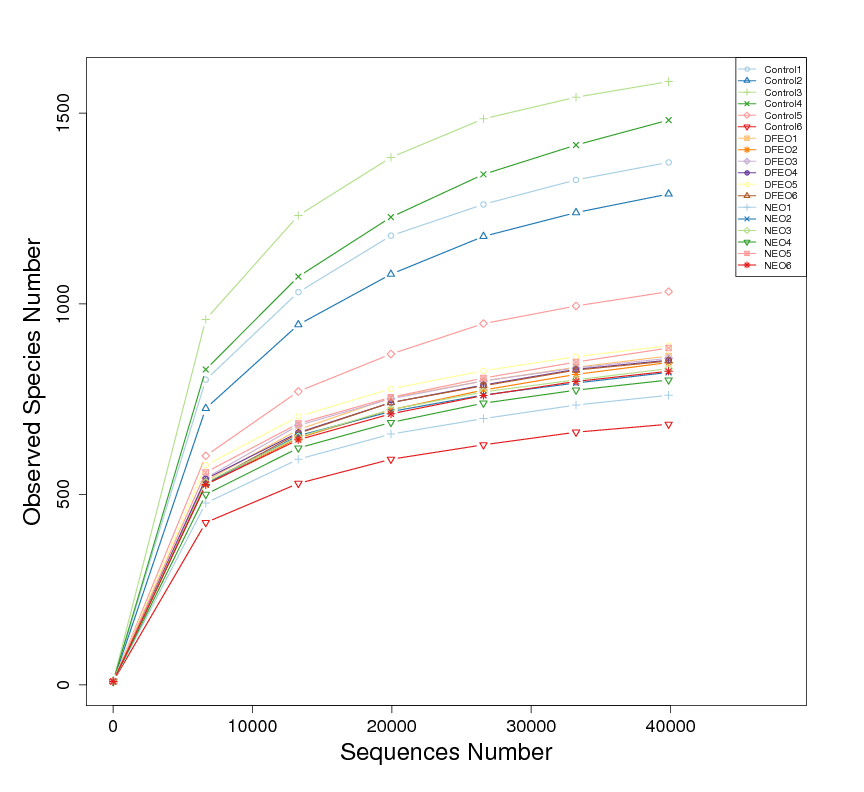


**Fig. S2.** Clustering and annotation of the OTUs of samples. The X-axis is different sample names. The first Y-axis is Tags number, and the second Y-axis is OTUs number.


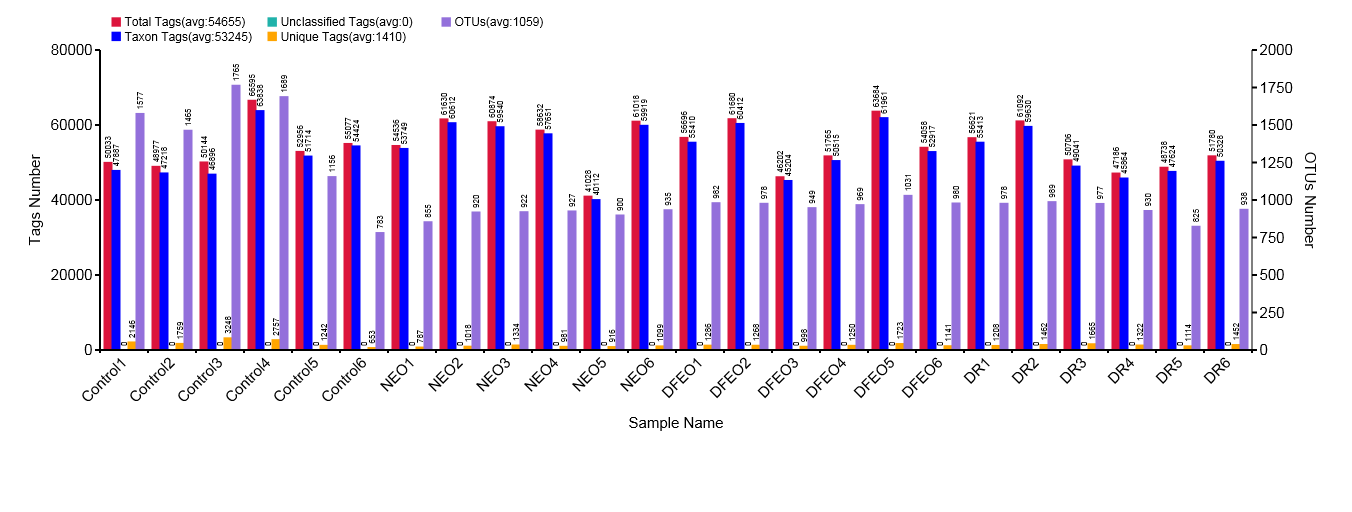


**Table S1. Components of basal diets**

| Ingredient | % |
| --- | --- |
| Casein | 20.0 |
| Lard | 3.0 |
| Soybean oil | 2.0 |
| Vitamin mixture^a^ | 1.0 |
| Mineral mixture^b^ | 4.0 |
| Choline chloride | 0.2 |
| Cellulose | 4.0 |
| Corn starch | 65.8 |
| Total | 100.0 |

^a^AIN-93: vitamin mixture; ^b^AIN-93: mineral mixture.

**Table S2** MRPP for difference among Control, NEO and DFEO groups

| Group | A | Observed-delta | Expected-delta | Significance |
| --- | --- | --- | --- | --- |
| Control-NEO | 0.03911 | 0.4397 | 0.4576 | 0.02 |
| Control-DFEO | 0.0613 | 0.4696 | 0.5003 | 0.008 |
| DFEO-NEO | 0.07342 | 0.4432 | 0.4783 | 0.003 |

Rats were fed with basal diet (control), basal diet plus unheated canola oil (NEO), and basal diet plus deep-fried canola oil (DFEO).
